# Effects of *Rhizopus oligosporus*-Mediated Solid-State Fermentation on the Protein Profile and α-Glucosidase Inhibitory Activity of Selenium-Biofortified Soybean Tempeh

**DOI:** 10.3390/foods14162899

**Published:** 2025-08-21

**Authors:** Chengying Wang, Changli Hu, Xin Li, Ruizhe Shen, Liwei Yin, Qiguo Wu, Ting Hu

**Affiliations:** 1 College of Life Science, Anqing Normal University, Anqing 246011, Chinalixinby2025@163.com (X.L.);; 2 Key Laboratory of Innovative Application of Characteristic Traditional Chinese Medicine Resources in Southwest Anhui Province of Anqing Medical College, Anqing 246052, China; 3 Key Laboratory of Biodiversity Conservation and Characteristic Resource Utilization in Southwest Anhui, Anqing 246011, China

**Keywords:** selenium, solid-state fermentation, tempeh, protein

## Abstract

Solid-state fermentation (SSF) enhances the nutritional profile of legumes. This study evaluated *Rhizopus oligosporus*-mediated SSF for selenium (Se) biofortification in soybean tempeh (a traditional Southeast Asian food), assessing the effects of selenate and selenite (0–60 mg kg^−1^) on *R. oligosporus* growth, substrate consumption, mycelium morphology, and Se speciation in tempeh. Selenium supplementation at 18–24 mg kg^−1^ reduced soybean protein content by 9.4~13.8% relative to the protein content of the Se-free fermented tempeh (control group, 19.85%) and significantly promoted proteolysis. Higher concentrations (48–60 mg kg^−1^) restored protein levels to control values (19%), indicating concentration-dependent regulation of protein stability. Selenate at 42 mg kg^−1^ significantly increased the levels of flavor amino acids (e.g., glutamate, aspartate), essential amino acids, and total amino acids in tempeh. In contrast, selenite showed no significant improvement in amino acid content and even reduced non-essential amino acids (e.g., alanine, glycine) at high concentrations (42 mg kg^−1^). Selenium biofortification converted selenate to selenomethionine (SeMet) and Se(VI), but transformed selenite into methylselenocysteine (MeSeCys), selenocystine (SeCys_2_), and SeMet. Fermented Se-tempeh demonstrated potent α-glucosidase inhibition (IC_50_ values ranging from 1.66 ± 0.05 to 2.89 ± 0.03 mg mL^−1^), suggesting Se-enriched soybean tempeh could be considered a promising blood-sugar-friendly food. Thus, developing soybean-based functional foods via co-inoculation of *R. oligosporus* with inorganic Se is a promising way to enhance tempeh bioactivity.

## 1. Introduction

Selenium (Se) is an essential trace element that is required for humans [1]. Insufficient Se intake is a global health concern, affecting an estimated 1 billion people worldwide [2]. A lack of adequate daily Se can lead to deficiency, with potential adverse health consequences. Se functions as a structural component of selenoproteins, including glutathione peroxidases and thioredoxin reductases, which are critical for maintaining redox homeostasis and modulating immune responses [3]. Thus, the integration of Se-enriched food into the diet represents a meaningful measure to avoid deficiency.

Solid-state fermentation (SSF) represents a promising strategy for enhancing the nutritional quality of legumes. Specifically, SSF using *Rhizopus oligosporus* offers a biologically efficient platform for Se biotransformation, while simultaneously mitigating the multiple nutritional constraints associated with soybean substrates. The genus of filamentous fungi, such as *Rhizopus*, is acknowledged as being GRAS (Generally Regarded as Safe) by the Food and Agriculture Organization of the United Nations (FAO). These fungi are primarily utilized in SSF, particularly in Asian nations, for the production of soybean-based foods such as tempeh [4]. The filamentous fungus not only secretes extracellular proteases and α-amylases [5] that hydrolyze macromolecular complexes but also demonstrates significant metallo-accumulation capacity, particularly for essential trace elements [6].

Previous studies have confirmed that fungi can accumulate metals and hydrolyze macromolecules [7]. Our research further shows that *R. oligosporus* has a remarkable ability. It can biotransform inorganic selenium into organic forms with better nutritional value. Analyses confirm that the fungus could mediated that bioconversion of inorganic selenite (SeO_3_^2−^) into organic Se compounds, predominantly selenomethionine (SeMet), through sulfur assimilation pathways [8,9]. Moreover, Se-enriched fungi may have a higher antioxidant capacity relative to conventional fungi [10]. Dietary sources rich in organic Se have been shown to sustain selenoenzyme activity for extended durations during Se depletion compared to those containing inorganic Se [11]. Natural Se-enriched sources primarily contain organic Se compounds [12]. Conversely, inorganic forms such as sodium selenite and sodium selenate, commonly used in dietary supplements, are not naturally occurring components of conventional diets [13]. Upon absorption, these inorganic Se species cannot be directly incorporated into systemic proteins [14]. The present investigation addresses the need for sustainable Se fortification strategies in fungal-based protein matrices. The experimental design incorporates three methodological innovations: (1) mycelial morphology and extension rates as a function of selenite and selenate concentrations; (2) quantification of protein and amino acid contents in fermented soybean under graded selenate and selenite treatments; and (3) speciation analysis via high performance liquid chromatography-hydride generation-atomic fluorescence spectrometry (HPLC-HG-AFS) to quantify Se biotransformation in soybean mediated by *R. oligosporus*. These approaches collectively advance our understanding of fungal-mediated Se metabolism in SSF systems while establishing scientifically validated parameters for producing nutritionally optimized, Se-biofortified tempeh. Therefore, this work provides a foundation for developing Se-biofortified tempeh as a functional food and for its potential use in targeted micronutrient intervention strategies.

## 2. Materials and Methods

### 2.1. Materials and Strains

All chemicals, including dextrose, MgSO_4_, KH_2_PO_4_, agar powder (Sinopharm Chemical Reagent Co., Ltd., Shanghai, China), sodium selenate, and sodium selenite (Sigma-Aldrich, St. Louis, MO, USA), were of analytical grade.Deionized water was used for all procedures. *R. oligosporus* (isolated from traditional tempeh, screened for protease activity, and taxonomically confirmed by ITS sequencing in our laboratory) was utilized for soybean fermentation. For strain activation, a mycelial inoculum was prepared by culturing *R. oligosporus* in potato dextrose broth [PDB, 20% (*w*/*v*) potato, 2% glucose] at 24 °C with 110 rpm agitation for 72 h until dense, white floccose mycelia developed.

### 2.2. Schematic Overview of the Experimental Program

The experimental program of this work was shown in Figure S1, the detailed experimental methods were described in the following sections.

### 2.3. Se-Enriched Culture and Growth Monitoring of R. oligosporus in Plate Assay

#### 2.3.1. Selenium Preparation and Media Formulation

Stock solutions of sodium selenate and sodium selenite were prepared in sterile deionized water at 10,000 μg mL^−1^ concentrations. These were aseptically added to potato dextrose agar [PDA, 20% (*w*/*v*) potato, 2% glucose, and 16% agar] to achieve final Se concentrations of 0 (control), 100, 200, 300, and 400 μg mL^−1^. Media were sterilized by autoclaving at 121 °C for 20 min and dispensed into 90 mm sterile petri dishes under laminar flow conditions. Quadruplicate plates were prepared for each treatment group.

#### 2.3.2. Fungal Cultivation and Growth Analysis

*R. oligosporus* was activated on PDA plates as previously described and inoculated onto a series of PDA plates with varying concentrations of selenate and selenite. Plates were incubated at 28 °C in darkness at 28 ± 0.5 °C with 70 ± 5% relative humidity. Colony diameter was measured daily using a caliper to assess the growth rate of *R. oligosporus* under varying Se concentrations. Concurrently, hyphal extension rates were modeled using kinetic parameters derived from daily growth measurements, enabling the prediction of optimal selenite and selenate concentrations for maximal fungal growth.

Colony diameters were measured at 24 h intervals for 5 days using a digital vernier caliper (Mitutoyo 500–196, ±0.01 mm precision). The radial growth rate (RGR) was calculated using the formula

$$\mathrm{RGR}\;(\mathrm{mm}\;{\mathrm{day}}^{-1})=\frac{\left(D_{2}-D_{1}\right)}{\left(t_{2}-t_{1}\right)}$$

where D_1_ and D_2_ represent colony diameters (mm) at times t_1_ and t_2_ (days), respectively. Initial inoculation size (5 mm) was subtracted from all measurements. Data represent mean values from four biological replicates.

### 2.4. Solid Fermentation

#### 2.4.1. Preparation of Selenium-Enriched Soybean Tempeh

Kaohsiung Number 9 soybeans were used for co-fermented tempeh. Soybeans were washed and soaked for 12 h and the outer membranes were removed. After drying, water (twice the weight of soybeans) was added to the soybeans, and the mixture was sterilized at 121 °C for 15 min. Filter-sterilized (0.45 μm PVDF) selenate and selenite were added to the culture medium to set the Se element concentration to 0, 6, 12, 18, 24, 30, 36, 42, 48, 54, and 60 mg kg^−1^.

#### 2.4.2. Inoculum Preparation of *Rhizopus oligosporus*

*R. oligosporus* was inoculated into soybean cereal for SSF to prepare *R. oligosporus*-fermented cereals. The detailed procedure is described as follows. The spore suspension of *R. oligosporus* (1 × 10^3^ spores mL^−1^) in sterile water was prepared according to the previously reported method [15]. The cereals (substrate) were sterilized at 121 °C for 30 min, inoculated with 20 mL *R. oligosporus* spore suspension, and fermented at 30 °C for 72 h under dark conditions. The fermented cereals were dried in a drying oven at 50 °C. Finally, the dried cereals were ground and passed through a 60-mesh sieve. The whole soybean meal was fermented by *R. oligosporus*, which served as the cereal substrate for edible fungus fermentation.

### 2.5. Dry Matter Content

The dry matter content was calculated from samples dried to a constant weight in a drying oven at 50 °C. Dry matter determination was performed in accordance with Association of Official Agricultural Chemists [16]. Percentage of consumed refers to the proportion of substrate utilized by microorganisms during fermentation relative to the initial total amount. The calculation formula is as follows:

$$\mathrm{Percentage}\;\mathrm{of}\;\mathrm{consumed}\;(\%)=\left(1\;-\;\frac{\mathrm{Residual}\;\mathrm{substrate}\;(g)}{\mathrm{Initial}\;\mathrm{substrate}\;(g)}\right)\;\times \;100\%$$

### 2.6. Determination of Amino Acids

Amino acid composition (except for the tryptophan) was measured using an L-8900 amino acid analyzer (Hitachi High-Technologies, Tokyo, Japan) after protein samples had been hydrolyzed with 6 M HCl at 110 °C for 24 h in a sealed tube, according to the method reported by Eveleigh [17].

### 2.7. Determination of Protein

Total protein content was quantified using the Kjeldahl method [16]. Briefly, accurately weighed samples (0.50 ± 0.01 g) were digested with concentrated H_2_SO_4_ (98%) and catalyst (K_2_SO_4_/CuSO_4_, 10:1 *w*/*w*) at 420 °C for 60 min. The liberated ammonia was steam-distilled into 40 g L^−1^ boric acid solution and titrated against 0.1 M HCl. Nitrogen content was converted to protein using a conversion factor of 6.25, based on the average 16% nitrogen content in soybean proteins. Each sample was analyzed in triplicate to ensure the accuracy and precision of the results.

### 2.8. Determination of Total Selenium

Total Se content was determined by hydride generation–atomic fluorescence spectrometry (HG-AFS) following acid digestion [18]. Homogenized samples (0.20 ± 0.01 g) were digested with 10 mL concentrated HNO_3_ (69%) in pre-cleaned Teflon tubes. After 24 h pre-digestion at room temperature, samples were heated at 180 °C for 2 h on a graphite hotplate. Digestates were cooled, diluted to 25 mL with ultrapure water, and filtered through 0.45 μm membranes. For Se(VI) reduction, 4 mL aliquots were mixed with 1 mL HCl (6 mol L^−1^) and heated at 99 °C for 60 min in a temperature-controlled water bath. All samples and reagent blanks were analyzed in triplicate using HG-AFS under optimized conditions: 1.0% KBH_4_ in 0.1% NaOH as reductant and carrier gas flow 400 mL min^−1^ Ar. The Se ion concentration in the digestive solution was determined using a standard curve method. Signal responses for Se and internal standard elements were recorded simultaneously.

Selenium bioconcentration factors (BCFs) were calculated as follows:

$$\mathrm{BCF}=\frac{\mathrm{Total}\;\mathrm{selenium}\;\mathrm{content}\;\mathrm{in}\;\mathrm{tempeh}\;({\mathrm{mg}\;L}^{-1})}{\mathrm{Added}\;\mathrm{selenium}\;\mathrm{concentration}\;\mathrm{in}\;\mathrm{fermented}\;\mathrm{substrate}\;({\mathrm{mg}\;L}^{-1})}\;\times \;100\%\;$$

### 2.9. Analysis of Selenium Speciation

Selenium species were extracted and quantified following a modified protocol [18]. Dried samples 0.100 ± 0.005 g were enzymatically hydrolyzed in 5 mL Tris-HCl buffer (5 mM, pH 7.5) containing 8 mg mL^−1^ Protease XIV (Sigma Chemical Co., St. Louis, MO, USA) at 37 °C for 24 h. After centrifugation (5000× *g*, 30 min, 4 °C), supernatants were filtered (0.22 μm nylon) and analyzed by ion-pair reversed-phase HPLC-HG-AFS (SA-20, Beijing Titan Instrument Co., Ltd., Beijing, China). Chromatographic separation utilized an anion-exchange column (Hamilton RPR-X100) (5 μm, 250 × 4.6 mm) with the mobile phase 40 mM (NH_4_)_2_HPO_4_ (pH 6.0) at a flow rate of 1 mL min^−1^. Peaks were identified according to the retention times of standard compounds [i.e., Se-methylselenocysteine (MeSeCys), selenocystine (SeCys_2_), Se (IV), SeMet, and Se (VI)] purchased from the National Research Center for Certified Reference Materials, Beijing, China. The identified Se species were quantified based on the peak areas of the calibration curves using an HPLC workstation.

### 2.10. In Vitro Digestion Model for α-Glucosidase Inhibitory Activity Testing of Tempeh

In vitro, the α-glucosidase (a digestive enzyme) inhibition assay was employed to determine the Se-enriched tempeh effectiveness. The simulated gastrointestinal phases followed the INFOGEST 2.0 static model [19]. Briefly, the INFOGEST 2.0 static digestion model was executed in three phases: (1) Oral phase (2 min, 37 °C), 5 g homogenized tempeh mixed 1:1 (*w*/*v*) with simulated salivary fluid (SSF) supplemented with α-amylase (75 U mL^−1^, pH 6.8); (2) Gastric phase (2 h, 37 °C), oral bolus combined 1:1 (*v*/*v*) with simulated gastric fluid (SGF) containing pepsin (2000 U mL^−1^, pH 3.0); (3) Intestinal phase (2 h, 37 °C), neutralized gastric chyme (pH 7.0) supplemented 1:1 (*v*/*v*) with simulated intestinal fluid, pancreatin (100 U mL^−1^ trypsin activity), and bile extract (10 mM). Digestion was terminated by ice immersion, followed by centrifugation (10,000× *g*, 30 min, 4 °C), filtration (0.22 μm PVDF), and storage at −80 °C. For the α-glucosidase inhibition assay, digestate (50 μL) was pre-incubated with enzyme solution (0.2 U mL^−1^, pH 6.8, 10 min, 37 °C), then reacted with pNPG substrate (50 μL, 5 mM) for 20 min. Reactions were terminated with Na_2_CO_3_ (100 μL, 0.2 M), and absorbance was measured at 405 nm. Acarbose was used as a positive control (10–1000 µg mL^−1^). The inhibitory activity of α-glucosidase was expressed as a percentage of inhibition according to the following formula:

$$\mathrm{Inhibition}\;(\%)\;=\frac{A-B}{C}\;\times \;100$$

where A is the absorbance of the sample, B is the absorbance of the sample blank, and C is the absorbance of the standard. The inhibition results are expressed as the mean inhibitory concentration (IC_50_), which is a measure of the efficacy of a compound in inhibiting biochemical function.

### 2.11. Statistical Analysis

Mean values and standard deviations (SD) were calculated using Microsoft Excel 2016. All data are provided as the mean ± SD. Graphs were generated using Origin Pro 2021 software (Origin Lab Corporation, Northampton, MA, USA). Significant differences were determined by one-way analysis of variance (ANOVA) followed by Duncan’s multiple-range test. Differences were considered significant at *p* < 0.05.

## 3. Results

To elucidate the dose–response effects of Se on *R. oligosporus* physiology during tempeh fermentation, graded Se concentrations (0–60 mg kg^−1^ substrate) were supplemented into solid-state cultures. The resultant fermented products were benchmarked against Se-free controls through comparative assessment of substrate utilization efficiency, amino acid metabolomic profiling, crude protein content, and Se content and species. Notably, Se fortification induced concentration-dependent modulation of both growth dynamics and biochemical transformation pathways, resulting in distinct metabolic distributions relative to conventional tempeh production.

### 3.1. Se-Enriched Growth of R. oligosporus in the Plate

**Figure 1:**
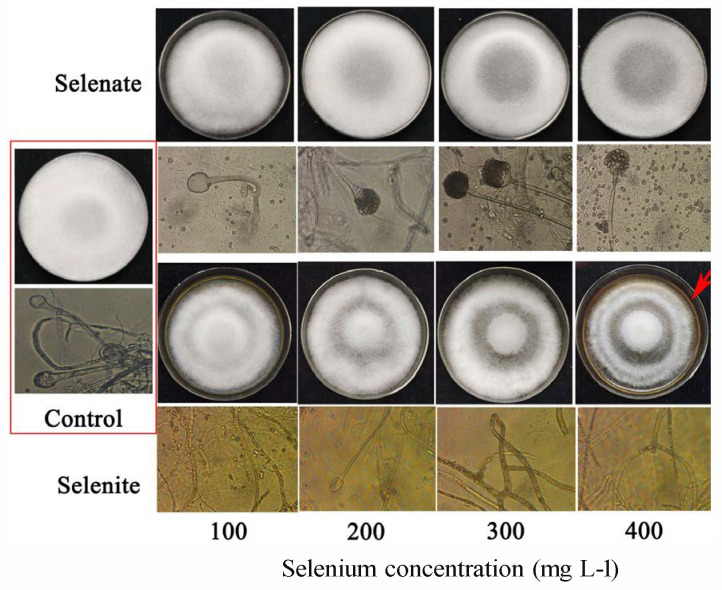
Influence of selenate and selenite on the colony and mycelium morphology of *R. oligosporus*. Note: the pink background in the plate containing > 400 mg L^−1^ selenium is due to the precipitation of elemental selenium.

The morphology analysis revealed that *R. oligosporus* exhibited distinct morphological responses to different Se species (Figure 1). Compared to the control group, the selenate-treated groups (100–400 mg L^−1^) exhibited a slight inhibitory trend on colony diameter. Sporulation showed a significant dose-dependent increase with rising selenate concentrations (Table S1). Similarly, selenite treatment demonstrated a non-significant reduction in colony diameter. Although colony diameter showed a decreasing tendency with increasing selenite concentrations, this effect was not statistically significant. Notably, hyphal thickness within colonies displayed a thinning trend as selenite concentrations increased. Both Se treatments significantly enhanced spore production, but selenite induced a distinctive “edge-initiated” distribution pattern (Table S1). Unlike the conventional center-radiating distribution, spore formation under selenite treatment initially occurred in the peripheral zone of colonies (first appearing 200–300 μm from the edge), representing a unique spatial sporulation phenotype.

### 3.2. Se-Enriched Growth of R. oligosporus in Soybean Tempeh

**Figure 2:**
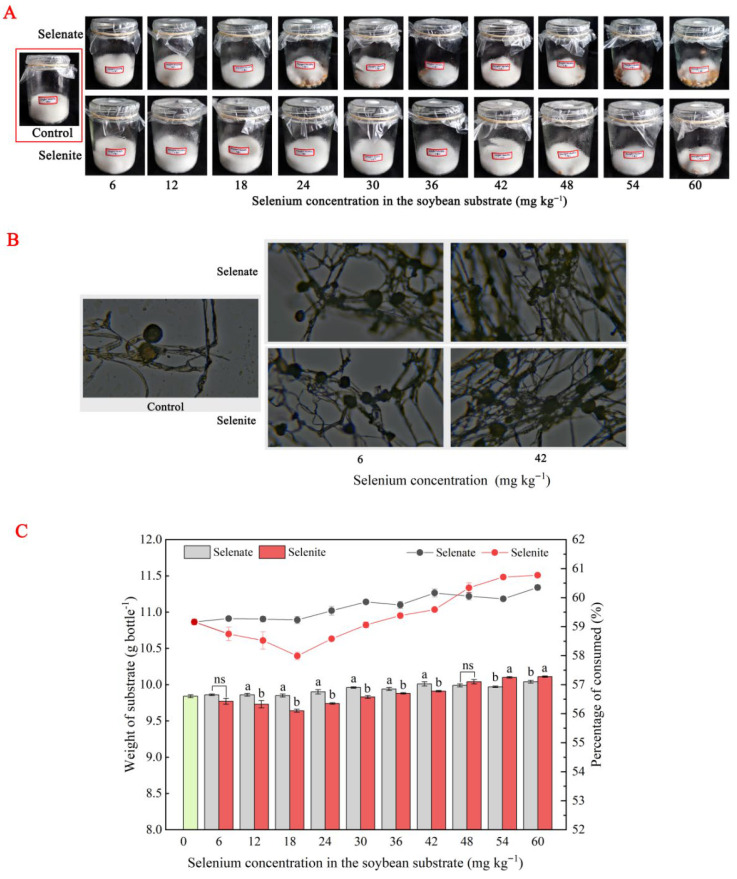
Effects of different concentrations of selenate and selenite on *R. oligosporus* metabolism after 3 days of incubation. (**A**) Fungal growth. (**B**) Microscopic photomicrographs during solid-state fermentation. (**C**) Substrate consumption. Note: *p* < 0.05 was considered as the significant level. Lowercase letters indicate statistically significant differences (*p* < 0.05) in protein content between selenate and selenite treatments at the same concentration; ns indicates no significant differences (*p* > 0.05).

Figure 2A demonstrates the robust growth of *R. oligosporus* on soybean substrates during SSF, characterized by the extensive development of dense, white mycelial networks. Microscopic examination revealed active reproductive activity, evidenced by prolific sporulation across the substrate surface. Analysis of the morphology, odor, and compactness data of tempeh fungal pellets during SSF showed that the mycelium grown by tempeh fungi treated with Se solution was thicker than that of the control group and more closely bound to the soybean matrix. The fungal pellets were lumpy and not easily loosened. However, when the Se concentration was too high, the mycelial distribution became sparse and loose. Microscopic observations revealed that colony colors ranged from light yellow to grayish-brown (Figure 2B). The mycelium exhibited typical morphological characteristics of *R. oligosporus*. Compared with the control group, the number of spores in the mycelium of the Se-treated groups increased significantly. Under SSF conditions, the mycelia in the control group exhibited robust growth with well-developed morphology and length, along with a relatively smooth surface (Figure S3A). Additionally, the hyphal tips formed intact, rounded conidia. These spherical structures displayed distinct connecting points and regular concentric protrusions. In contrast, the Se-treated groups showed noticeable alterations in mycelial morphology, including shrinkage and deformation. The hyphal tips appeared wrinkled and fragmented, with some chlamydospores observed at the tips. Conidia were widely distributed across the mycelial surface, but the connecting points appeared collapsed and wrinkled, with blurred outlines. Compared to the control group, the Se-treated mycelia underwent significant changes, such as contraction and distortion. Furthermore, the Se-treated groups exhibited a marked increase in spore production, generating a substantial number of conidia.

Substrate consumption rates showed a clear valence-state dependence. When the selenate concentration ranged from 0 to 60 mg kg^−1^, the substrate consumption rate of the selenate treatment groups showed an overall upward trend. In contrast, the substrate consumption rate of the sodium selenite groups exhibited a trend of first decreasing and then increasing. When the selenite concentration was 0–18 mg kg^−1^, the substrate consumption rate decreased; when the concentration was 18–60 mg kg^−1^, the consumption rate increased. At Se concentrations of 0–42 mg kg^−1^, the substrate consumption rate of the selenate groups was higher than that of the selenite groups; at 48–60 mg kg^−1^, the consumption rate of the selenate groups was lower than that of the selenite groups (Figure 2C). Energy-dispersive X-ray spectroscopy (EDX) analysis confirmed the incorporation of Se into the mycelia, with Se accounting for 23.7 ± 9.2% and 15.0 ± 9.3% of the elemental composition in sodium selenate- and selenite-treated samples, respectively (Figure S3B).

### 3.3. Effects of Se on the Protein and Amino Acid Contents in Soybean Tempeh

**Figure 3:**
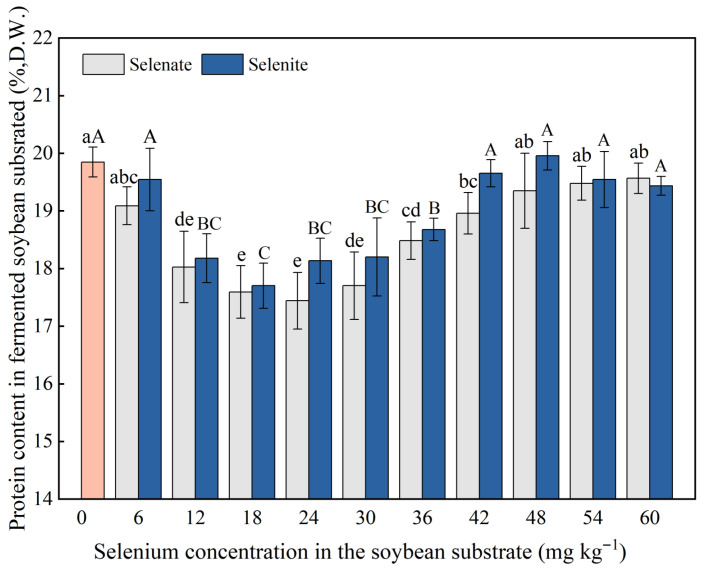
Effects of *R. oligosporus* and selenium treatment on the protein content of tempeh after solid-state fermentation. Vertical bars indicate standard deviation (*n* = 3, where *n* represents the number of replicates). *p* < 0.05 was considered as the significant level. Different lowercase letters indicate statistically significant differences (*p* < 0.05, ANOVA) in protein content across selenate concentration gradients. Different capital letters indicate statistically significant differences (*p* < 0.05, ANOVA) in protein content across selenite concentration treatments.

The results of protein content determination indicate that the protein content in fermented soybean tempeh can reach 19%, which is lower than that of natural soybeans, which is about 35% (Figure 3). The protein content is up to 19.85% in the control group without Se. When the selenate concentration was 6 mg kg^−1^, the protein content decreased to 19.09%, while the amino acid content increased significantly. The protein content further decreased to approximately 18.96% when the selenate concentration was 42 mg kg^−1^. The protein content decreased to less than that of the control group, and the amino acid content did not increase significantly at selenite concentrations of 6 mg kg^−1^ and 42 mg kg^−1^ (Table 1). According to the analysis of the results, Se can improve the activity of protein hydrolase and promote the hydrolysis of protein into peptides and amino acids, which facilitates absorption and utilization by the human body. Among these, the promoting effect of selenate on protein hydrolase activity was more significant than that of selenite, especially at the concentration of 42 mg kg^−1^, where the effect of protein hydrolysis was the most pronounced.

As shown in Table 1, the contents of flavor amino acids (FAA), essential amino acids (EAA), non-essential amino acids (NAA), and total amino acids (TAA) were determined. Glutamic acid was the most abundant amino acid in tempeh, followed by aspartic acid, phenylalanine, alanine, glycine, and tyrosine. Sodium selenate treatment significantly increased the contents of FAA, EAA, NAA, and TAA, and the effect was the most pronounced at 42 mg kg^−1^. In contrast, the amino acid content in the selenite treatment group was almost the same as that of the control group, and some indicators were slightly lower than those of the control group.

### 3.4. Effects of Se-Enriched SSF on the Se Content and Species

**Figure 4:**
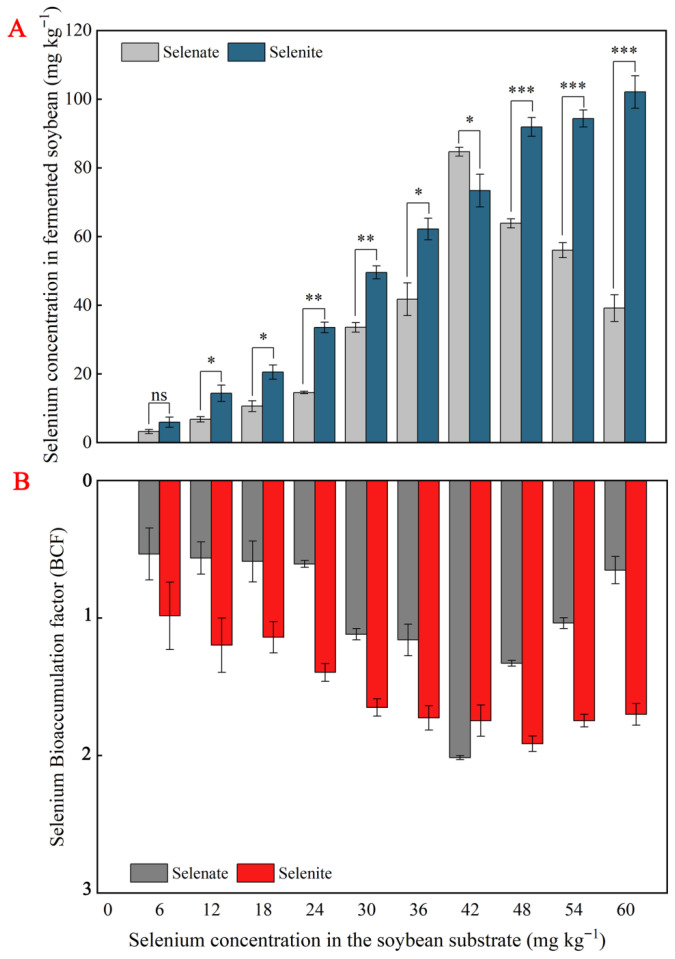
Selenium accumulation (**A**) and bioaccumulation factor (BCF) (**B**) in tempeh under selenate and selenite treatments during solid-state fermentation. Data represent mean values (*n* = 3, where *n* represents the number of replicates) ± standard deviation (SD). Asterisks denote significant differences compared to the control: *, *p* < 0.05; **, *p* < 0.01; ***, *p* < 0.001.

The results shown in Figure 4 demonstrate that a clear dose–response relationship was observed between Se application gradients and tempeh Se content. The Se content in tempeh samples exhibited an initial increase, followed by a decrease with increasing selenate concentration. Within the range of 0–42 mg kg^−1^, the Se content showed an upward trend, peaking at 42 mg kg^−1^. When the concentration increased to 42–60 mg kg^−1^, the Se content displayed a downward trend. The variation trend of the Se bioconcentration factor (BCF) was consistent with that of total Se content, also reaching its peak at 42 mg kg^−1^. In contrast, the Se content demonstrated an overall increasing trend with rising selenite concentration, attaining its maximum at 60 mg kg^−1^. However, the BCF exhibited an initial increase followed by a decrease, peaking at 48 mg kg^−1^. At the same concentration, the Se content in selenite-treated samples was generally higher than that in selenate-treated samples, except at 42 mg kg^−1^, where selenate treatment resulted in a higher Se content than selenite.

**Figure 5:**
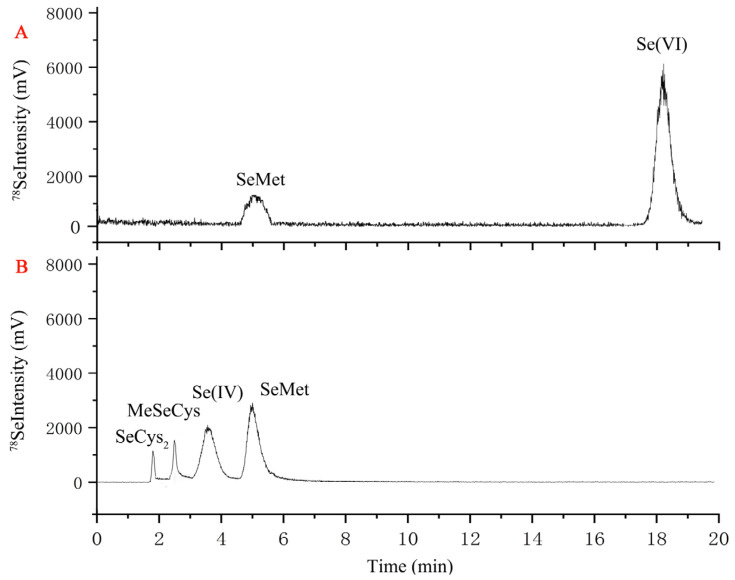
Examples of chromatograms of selenium (Se) speciation in protease XIV extracts of selenate- (**A**) and selenite-fermented (**B**) soybean, as determined by anion exchange HPLC-HG-AFS. The intensity (count per second, mV) is for m/z 78. Note: selenocysteine, SeCys_2_; selenomethylselenocysteine, MeSeCys; selenite, Se(IV); selenomethionine, SeMet; selenate, Se(VI).

Selenium species in the Se-biofortified tempeh were extracted and analyzed by enzymatic hydrolysis. All extraction efficiency rates were above 70.8%. Four Se species (SeCys_2_, MeSeCys, Se (IV), and SeMet) were observed in the 42 mg kg^−1^ selenite-treated samples, while two Se species (SeMet and Se (VI)) were observed in the samples treated with 42 mg kg^−1^ selenate (Figure 5). The predominant Se species in selenate-biofortified fruiting bodies were SeMet 21.4 ± 2.2% and Se (VI) 78.6 ± 5.0%. The proportion of organic Se was 84.1%, and the proportions of SeMet, SeCys_2_, and MeSeCys were 56.2 ± 4.1%, 20.4 ± 1.2%, and 7.5 ± 0.8% with selenite treatment (Figure 5; Table 2).

### 3.5. α-Glucosidase Inhibitory Activity of Selenium-Enriched Tempeh

**Figure 6:**
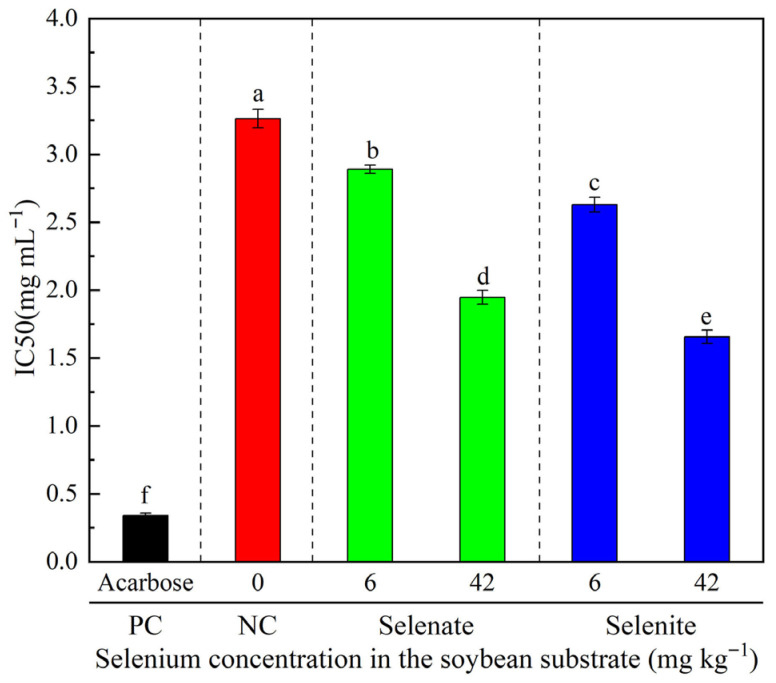
Mean inhibitory concentration (IC_50_) values of different treatments against α-glucosidase activity. Data are presented as mean ± SD (*n* = 3, where *n* represents the number of replicates). Bars labeled with different lowercase letters indicate significant differences among groups (ANOVA, *p* < 0.05). Acarbose was used as a positive control.

The Se-fermented tempeh exhibited potent α-glucosidase inhibitory activity, with IC_50_ values ranging from 1.66 ± 0.05 to 2.89 ± 0.03 mg mL^−1^ (all below 3.00 mg mL^−1^), which were significantly lower than that of Se-free fermented tempeh (3.26 ± 0.07 mg mL^−1^) (Figure 6). Moreover, the IC_50_ value for 42 mg kg^−1^ Se-fermented tempeh was significantly lower compared than that for 6 mg kg^−1^ Se-fermented tempeh. This indicates that Se-fermented tempeh has strong potential for hyperglycemia control compared with no-Se tempeh. The IC_50_ value of acarbose (0.34 ± 0.02 mg mL^−1^) indicates that Se potently suppressed the activity of this enzyme and could be considered a promising blood-sugar-friendly food.

## 4. Discussion

### 4.1. Selenium Supplementation Significantly Alters the Growth and Morphology of R. oligosporus

Tempeh is a traditional fermented soybean product; it is made by a natural fermentation process in which whole soybeans are inoculated with *R. oligosporus*, forming a compact, cake-like block. The present study demonstrates that Se supplementation significantly influences the growth dynamics and morphology of *R. oligosporus* mycelium (Figure 1 and Figure S2). Our findings align with previous reports indicating that Se can modulate plant and fungal physiology in a dose- and valence-dependent manner [20,21]. The observed biphasic response, where low Se levels stimulate fungal growth and metabolic activity while higher concentrations induce inhibitory effects, is consistent with the hormesis phenomenon observed in other Se-enriched microbial systems [22]. The morphological alterations in Se-treated mycelia, including hyphal shrinkage, increased sporulation, and disrupted conidial structures, may reflect Se-induced oxidative stress or interference with cell wall biosynthesis. Selenium primarily modulates the cell wall architecture by regulating the activity and expression of enzymes involved in polysaccharide synthesis and cross-linking. Specifically, it suppresses the synthesis of hemicellulose I while promoting the deposition of hemicellulose II, as exemplified in the roots of *Brassica rapa* under cadmium stress [23], a mechanism that may be conserved in fungal cell wall regulation. In *Saccharomyces boulardii*, González-Salitre observed a 112% increase in Sec-GPx activity and an 89% elevation in TrxR activity, which collectively potentiated H_2_O_2_ and lipid peroxide scavenging capacity in the fungal system [24]. Beyond antioxidant defense, selenite pressure regulates the biosynthesis of secondary metabolites in Monascus [25]. Conversely, excessive Se induces oxidative stress in fungal hyphae by interfering with glutathione homeostasis, triggering a reactive oxygen species outburst and causing direct damage from Se species transformation products [18]. This oxidative damage may be attributed to strain aging, the evidence for which lies in the declining capacity to maintain organic Se accumulation during culture degradation [26]. These results demonstrate that *R. oligosporus* possesses significant inorganic Se bioaccumulation capacity and that its Se assimilation and transformation efficiency exhibits concentration-dependent enhancement. Similar deformities have been reported in *Aspergillus* spp. under Se exposure, where excessive Se incorporation disrupted redox homeostasis and cytoskeletal organization [27]. The elevated spore production in Se-treated groups could represent a fungal survival strategy, as sporulation is often upregulated under stress conditions to enhance dispersal and reproductive success [28]. The “edge-initiated” sporulation pattern under selenite treatment is particularly intriguing and warrants further investigation into potential Se-mediated signaling pathways affecting fungal developmental asymmetry.

### 4.2. Selenium Fortification Differentially Enhanced Tempeh Quality

Nutritionally, Se fortification significantly enhanced protein hydrolysis and amino acid liberation in Se-treated tempeh. Similarly, in alfalfa silage, Se enrichment significantly increased crude protein, soluble carbohydrate, total Se, and organic Se contents, while reducing neutral detergent fiber and acid detergent fiber levels [29]. In tempeh, sodium selenate is more effective than sodium selenite in promoting amino acid accumulation, likely due to its superior activation of protein hydrolase, which enhances the hydrolysis of proteins into amino acids. As shown in Figure 2B, Se treatments altered mycelial morphology (e.g., thicker hyphae at optimal concentrations; shrinkage/deformation at high levels) and increased sporulation. These structural changes correlate with elevated protein hydrolase activity, particularly in selenate-treated groups. Thicker mycelia (vs. control) likely provide a greater surface area for enzyme secretion, accelerating proteolysis. This explains the significantly higher amino acid contents (free, essential, non-essential, and total) in selenate groups, with peak efficacy at 42 mg kg^−1^ (Table 1). In contrast, excessive Se concentrations produced sparse mycelia, reducing substrate binding and enzyme efficiency—aligning with diminished protein hydrolysis under high-Se stress. This supports recent findings that Se modulates protease activity in filamentous fungi, potentially via enzyme conformational shifts or redox-sensitive regulation [21,30].

The differential Se accumulation and speciation patterns between selenate and selenite treatments have critical implications for Se-biofortified food production [8,31]. Peak Se content at 42 mg kg^−1^ (selenate) and 60 mg kg^−1^ (selenite), indicating valence-specific bioaccumulation thresholds, was possibly linked to cellular detoxification mechanisms such as volatilization or sequestration [32]. Selenite passively enters cells via silicon transporters and non-specific phosphate permeases, enabling rapid intracellular accumulation [33]. In contrast, selenate requires active uptake through sulfate transporters, competing with endogenous sulfate ions [34]. This competition limits selenate assimilation—particularly in sulfate-rich substrates like soybeans. Consistent with this mechanism, our previous study on Se-enriched *Auricularia auricula* demonstrated that, at equivalent concentrations, selenite achieved 2.3-fold higher bioaccumulation than selenate—an outcome attributed to reduced transporter competition [35]. Consequently, selenite-treated tempeh exhibited higher total Se and superior organic Se conversion efficiency. This supports selenite’s preferential use in functional foods, aligning with consumer demand for bioactive Se compounds [36].

The higher proportion of organic Se species (SeMet, SeCys_2_, MeSeCys) in selenite-treated tempeh is nutritionally advantageous, as these forms exhibit superior bioavailability and antioxidant properties compared to inorganic Se [37]. The dominance of SeMet in selenite-treated samples suggests that *R. oligosporus* efficiently incorporates selenate into methionine analogs, a process facilitated by the sulfur assimilation pathway [38]. Selenium has fermentation properties and biological activity in addition to acting as an organic carrier of Se [39]. Organic Se has higher bioavailability, is easier for the body to absorb, and is used for its physiological function. Future studies should explore genetic engineering approaches to further optimize Se metabolism in *R. oligosporus* for enhanced nutritional benefits.

### 4.3. Se-Fermented Tempeh Significantly Enhanced α-Glucosidase Inhibitory Activity

Se-fermented tempeh exhibited significantly enhanced α-glucosidase inhibitory activity, with an IC_50_ value of <3.00 mg mL^−1^ versus 3.26 ± 0.07 mg mL^−1^ in Se-free tempeh. This approximate 9.4~13.8% reduction in IC_50_ highlights Se enrichment’s critical role in boosting the substrate’s antidiabetic potential. Given that α-glucosidase inhibition directly targets carbohydrate hydrolysis in the small intestine—delaying glucose absorption and serving as a key anti-hyperglycemic indicator [40]—these findings demonstrate functional enhancement. Notably, our Se-enriched tempeh achieved stronger inhibition than that reported for other substrates. Hossain et al. [41,42] observed IC_50_ values of 3.87 mg mL^−1^ for Koda and 4.97 mg mL^−1^ for Kissendrup, respectively. This reduction in IC_50_ signifies a substantial improvement in inhibitory potency, directly correlating with Se’s ability to modulate enzymatic activity [43]. This enhanced inhibition may arise from Se’s integration into tempeh’s protein matrix during fermentation, forming seleno-amino acids (e.g., selenomethionine) or selenopeptides that competitively bind to α-glucosidase’s active site. Furthermore, the dose-dependent efficacy—where tempeh fermented with 42 mg kg^−1^ Se demonstrated significantly stronger inhibition than the 6 mg kg^−1^ Se variant—underscores Se concentration as a key determinant in optimizing α-glucosidase suppression. Selenium could potentiate the activity of inherent bioactive compounds (e.g., phenolics, peptides) in plants and fungi via synergistic interactions [22,44]. This indicated that Se-enriched tempeh has the capacity to enhance antidiabetic properties through enzymatic modulation. Notably, while Se-fermented tempeh exhibits promising activity, its IC_50_ remains higher than that of the pharmaceutical control acarbose (0.34 ± 0.02 mg mL^−1^). This disparity is expected, given acarbose’s specificity as a potent α-glucosidase inhibitor. Previous research demonstrated that Se-fermented foods represent a novel pharmacological intervention for prediabetic populations [45]. Thus, the bioactivity of Se-tempeh positions it as a compelling natural alternative for glycemic management, particularly due to its dual function as a functional food that delivers essential micronutrients (e.g., organic Se) while also serving as an effective adjunct for glycemic control.

## 5. Conclusions

This study demonstrates that Se fortification significantly influences *R. oligosporus* growth, morphology, and metabolic activity during tempeh fermentation. Selenate and selenite exhibited valence-specific effects: selenate transiently promoted colony expansion at 200 mg L^−1^ but reduced hyphal thickness, while selenite delayed early growth yet enhanced sporulation with a unique “edge-initiated” pattern. Optimal Se enrichment occurred at 42 mg kg^−1^ (selenate) and 60 mg kg^−1^ (selenite), with selenate more effectively boosting amino acid release via protease activation. Notably, selenite favored organic Se conversion (84.1%, mainly SeMet and SeCys_2_), enhancing nutritional bioavailability. These findings highlight *R. oligosporus* as a promising biofactory for Se-biofortified foods through excessive Se-induced oxidative stress altering mycelial structure. Future research should optimize Se metabolism pathways to maximize organic Se yield while minimizing fungal stress and advancing functional food development.

## Figures and Tables

**Table 1:** Amino acid content in tempeh with selenium treatments.

Amino Acid (%)		Control	Selenate (mg kg^−1^)		Selenite (mg kg^−1^)	
			6	42	6	42
Flavor amino acid	Glutamic acid	6.43 ± 0.09 c	7.21 ± 0.09 b	7.54 ± 0.04 a	6.50 ± 0.01 c	6.54 ± 0.01 c
	Aspartic acid	4.35 ± 0.03 d	4.60 ± 0.01 b	4.74 ± 0.03 a	4.40 ± 0.02 c	4.41 ± 0.01 c
	Phenylalanine	2.09 ± 0.01 bc	2.13 ± 0.02 a	2.07 ± 0.02 c	2.10 ± 0.01 b	2.11 ± 0.01 ab
	Alanine	2.47 ± 0.09 a	2.41 ± 0.02 ab	2.06 ± 0.01 b	2.36 ± 0.07 c	2.17 ± 0.02 d
	Glycine	1.66 ± 0.01 c	1.69 ± 0.01 b	1.73 ± 0.01 a	1.65 ± 0.01 c	1.63 ± 0.01 d
	Tyrosine	1.58 ± 0.02 a	1.55 ± 0.04 a	1.57 ± 0.03 a	1.57 ± 0.01 a	1.58 ± 0.01 a
	Total	18.58 ± 0.02 c	19.59 ± 0.09 b	19.71 ± 0.09 a	18.57 ± 0.06 c	18.43 ± 0.02 d
Essential amino acids		13.69 ± 0.04 b	14.71 ± 0.10 a	14.74 ± 0.10 a	13.63 ± 0.03 bc	13.55 ± 0.03 c
Non-essential amino acid		22.16 ± 0.13 c	23.74 ± 0.21 b	24.35 ± 0.03 a	22.28 ± 0.07 c	22.20 ± 0.01 c
Free amino acids		30.44 ± 0.05 c	32.50 ± 0.21 b	33.10 ± 0.09 a	30.49 ± 0.03 c	30.32 ± 0.04 c
Total amino acids		35.86 ± 0.09 c	38.45 ± 0.31 b	39.09 ± 13 a	35.92 ± 0.07 c	35.76 ± 0.04 c

Data represent mean ± SD (*n* = 3, where *n* represents the number of replicates). Different lowercase letters indicate statistically significant differences between treatments (ANOVA, *p* < 0.05).

**Table 2:** Percentage of selenium speciation in tempeh fermented with 42 mg kg^−1^ selenium.

Treatment	Selenium Speciation (%)				
	SeCys_2_	MeSeCys	Se(IV)	SeMet	Se(VI)
Selenate	/	/	/	21.4 ± 2.2	78.6 ± 5.0
Selenite	20.4 ± 1.2	7.5 ± 0.8	15.9 ± 0.3	56.2 ± 4.1	/

Selenocysteine, SeCys_2_; selenomethylselenocysteine, MeSeCys; selenite, Se(IV); selenomethionine, SeMet; selenate, Se(VI). “/” indicates values below the detection limit.

## Data Availability

The original contributions presented in the study are included in the article and Supplementary Materials, further inquiries can be directed to the corresponding author.

## Supplementary Materials

The following supporting information can be downloaded at: https://www.mdpi.com/article/10.3390/foods14162899/s1, Figure S1: Flow-sheet of selenium enriched tempeh production; Figure S2: Dynamic changes in mycelial biomass, colony diameter, and growth rate of *Rhizopus oligosporus* under various concentrations of selenate (A) and selenite (B) treatments; Figure S3: SEM images of tempeh in selenate and selenite treatments (A), and EDX spectra of the 42 mg kg^−1^ selenate and selenite treatments (B); Table S1: Spore Count of *Rhizopus oligosporus* in different selenium treatments. Note: + indicates 10^6^–10^7^CFU/plate, ++ indicates 10^8^–10^9^ CFU/plate, +++ indicates 10^9^–10^10^ CFU/plate.
