# Supplementary material for: Effects of Rhizopus oligosporus-Mediated Solid-State Fermentation on the Protein Profile and α-Glucosidase Inhibitory Activity of Selenium-Biofortified Soybean Tempeh
Source: Foods. 2025 Aug 21;14(16):2899. doi: 10.3390/foods14162899 (PMC12385420; doi:10.3390/foods14162899)
Supplement: Supplementary file 1 [file foods-14-02899-s001.zip › foods-3772569-supplementary.pdf]

Supplement materials

Figure S1 Flow-sheet of selenium enriched tempeh production.

Figure S2 Dynamic changes in mycelial biomass, colony diameter, and growth rate of *Rhizopus oligosporus* under various concentrations of selenate (A) and selenite (B) treatments.

Figure S3 SEM images of tempeh in selenate and selenite treatments (A), and EDX spectra of the 42 mg kg^{-1} selenate and selenite treatments (B).

Table S1 Spore Count of *Rhizopus oligosporus* in different selenium treatments. Note: + indicates 10^6–10^7CFU/plate, ++ indicates 10^8–10^9 CFU/plate, +++ indicates 10^9–10^{10} CFU/plate.

Figure S1

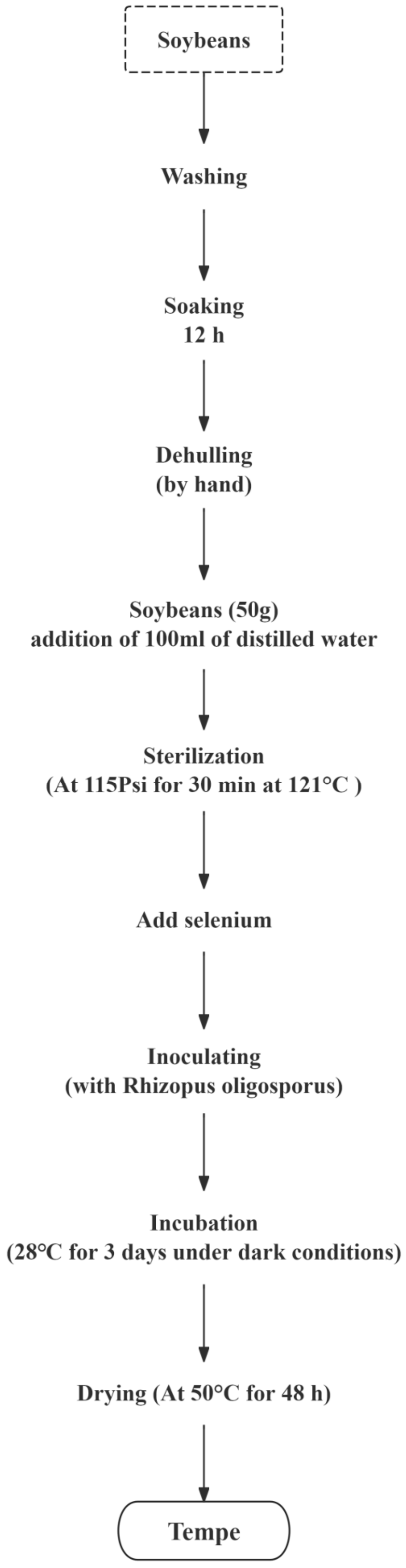
Soybeans
Washing
Soaking
12 h
Dehulling
(by hand)
Soybeans (50g)
addition of 100ml of distilled water
Sterilization
(At 115Psi for 30 min at 121°C)
Add selenium
Inoculating
(with Rhizopus oligosporus)
Incubation
(28°C for 3 days under dark conditions)
Drying (At 50°C for 48 h)
Tempe

Figure S2

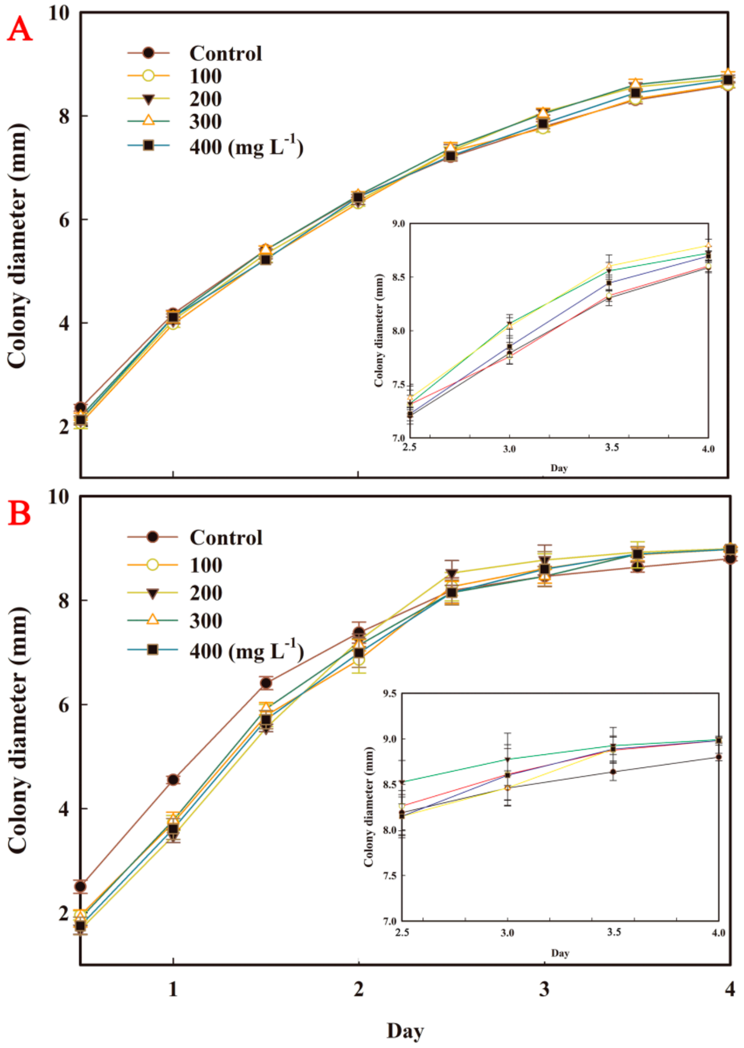

Figure S3

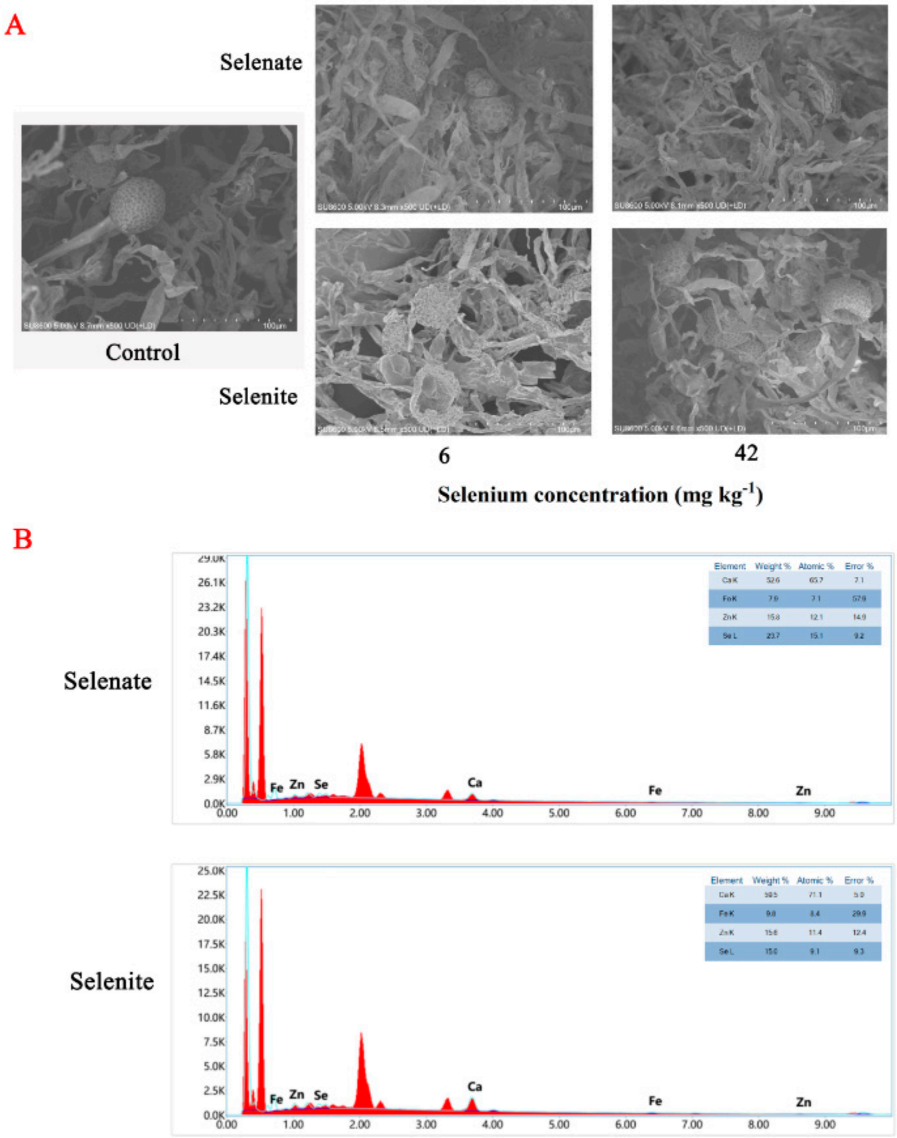
A
Selenate
Control
Selenite
6
42
Selenium concentration (mg kg-1)
B
Selenate
Element
Weight %
Atomic %
Error %
CaK 52.6 65.7 7.1
ZnK 15.8 12.1 14.9
Se L 23.7 15.1 9.2
Fe Zn Se
Ca
Fe
Zn
Selenite
Element
Weight %
Atomic %
Error %
Fe Zn Se
Ca
Fe
Zn

Table S1 Spore Count of *Rhizopus oligosporus* in different selenium treatments

Treatment	0 mg L^{-1}	100 mg L^{-1}	200 mg L^{-1}	300 mg L^{-1}	400 mg L^{-1}
Selenate	+	++	+	+++	+++
Selenite	+	++	+	+++	+++

Note: + indicates 10^6–10^7CFU/plate, ++ indicates 10^8–10^9 CFU/plate, +++ indicates 10^9–10^{10} CFU/plate.
